# Decontamination Validation of the BSL-4 Chemical Disinfectant Deluge Shower System

**DOI:** 10.1089/apb.2024.0011

**Published:** 2024-12-16

**Authors:** Anders Leung, Todd Cutts, Jay Krishnan

**Affiliations:** National Microbiology, Public Health Agency of Canada, Winnipeg, Canada.

**Keywords:** BSL-4, BSL-4 chemical shower, chemical shower validation, decontamination validation, surrogate virus, vesicular stomatitis virus

## Abstract

**Introduction:**

Positive pressure breathing-air-fed protective suits are used in biosafety level 4 (BSL-4) containment laboratories as personal protective equipment to protect workers from high-consequence pathogens. However, even with the use of primary containment devices, the exterior surfaces of these suits could potentially become contaminated with those pathogens and result in their inadvertent removal from containment. To address the risk of such pathogens escaping from containment via contaminated protective suits, these suits are decontaminated in a disinfectant chemical shower situated in an anteroom prior to exiting the BSL-4 laboratory. Properly diluted chemical disinfectants such as Micro-Chem Plus™ (MCP) or peracetic acid are used for this purpose. However, whether these suits are properly decontaminated during the chemical shower process needs to be validated.

**Methods:**

The purpose of this study was to develop a suit decontamination validation method for the BSL-4 chemical showers using a risk group 2 (RG2) surrogate virus for the high consequence pathogens that are handled in the BSL-4 laboratories. Here, we evaluated the efficacy of a 5% MCP shower using coupons made from different parts of protective suits (suit fabric, visor, boot, vinyl tape) laden with a dried-on mixture of vesicular stomatitis virus in tripartite organic soil load.

**Discussion:**

This validation study demonstrated that a chemical deluge shower procedure using 5% MCP for 2 min followed by a 3-min water rinse was successful in decontaminating the positive pressure suits that were experimentally contaminated with the live RG2 virus. This offers valuable insights into the rigor of the decontamination process being undertaken in the BSL-4 laboratory chemical showers.

## Introduction

Biosafety level 4 (BSL-4) laboratories operate at maximum biocontainment^1^ in order to conduct research and diagnostic work with high-consequence risk group 4 (RG4) pathogens.^2^ There are two types of BSL-4 laboratories: cabinet laboratories, where pathogens are handled in a line of class III biosafety cabinets, and suit laboratories, where personnel wear positive pressure breathing-air-fed protective suits^3^ while working in the laboratory. Cabinet laboratories can be ergonomically challenging for certain tasks and are not generally suitable for large animal work.^4^ As a result, the majority of BSL-4 laboratories are suit laboratories,^5,6^ where the workers enjoy enhanced range of motion and visibility compared with those working in the cabinet laboratories.

The suit laboratories are engineered, designed, built, commissioned, and operated to prevent the escape of high-consequence pathogens from BSL-4 containment. Some of the engineering features include dedicated HEPA filtered supply and exhaust air systems, airtight doors, and inward negative-pressure gradients across airtight doors to prevent the potentially contaminated air of the BSL-4 laboratory suite from flowing into adjacent areas. Additionally, decontamination equipment and strict protocols for decontaminating solid and liquid wastes^7^ are in place to ensure pathogens are properly inactivated prior to waste removal from the laboratory. Standard operating procedures, developed based on in-house validation data and local risk assessments, are strictly followed to further minimize the chances of pathogen escape from containment. Unlike a cabinet laboratory, the pathogens are not handled in fully enclosed class III biosafety cabinets in a suit laboratory; therefore, the suit’s external surfaces could become potentially contaminated with infectious materials, especially after a spill incident or while working in a cubicle housing experimentally infected animal. Even in the absence of an incident, all personnel leaving BSL-4 containment are required to decontaminate their suits using a chemical disinfectant shower, situated in an anteroom to the main laboratory on their normal exit route.

Different chemical disinfectants are used in the BSL-4 chemical shower; the most widely used disinfectant, particularly in the United States, Canada, Australia, and Asian laboratories,^6,8^ is Micro-Chem Plus™ (National Chemical Laboratories, Inc., Philadelphia, Pennsylvania). Micro-Chem Plus™ (MCP) is a quaternary ammonium compound (QAC) based detergent disinfectant; QAC-based disinfectants have shown microbicidal activities against a number of bacterial, viral, and fungal agents.^9^ In addition to two QACs (alkyl dimethyl ethylbenzyl ammonium chloride, alkyl dimethyl benzyl ammonium chloride), MCP also contains nonylphenol ethoxylates as surfactants.^10^ Nonylphenol ethoxylates and its biodegradation products are highly toxic to the aquatic environment;^11,12^ hence, the European Union has restricted the levels of nonylphenol ethoxylates in certain products marketed in European countries.^13,14^ As a result, certain BSL-4 laboratories in Europe use liquid peracetic acid (PAA) as an alternate, environmentally friendly chemical disinfectant in their chemical shower system instead of MCP.^15^

The use of QAC-based disinfectants in BSL-4 laboratories and their chemical showers was based on initial validation studies done mostly at the U.S. Army Medical Research Institute of Infectious Diseases (USAMRIID), Fort Detrick. Unfortunately, a majority of the early studies done at the USAMRIID were never published, but data presented at the 1982 American Biological Safety Association conference in Boston, MA (*Selection of Disinfectants for Inactivation of Lassa Virus under Field and Maximum Containment Laboratory Conditions*, P.B. Jahrling and A.I. Kuehne) showed inactivation of 6 logs of Lassa virus (suspended in 10% fetal bovine serum) in 5 min, when treated with either Steri Plus II or Roccal*,* two QAC-based disinfectants.

National Chemical Laboratories, the manufacturer of MCP, recommends a concentration of 1.56% (2 ounces per gallon of water) and 10 min of contact time for virucidal applications. However, most BSL-4 laboratories use MCP at higher concentrations, typically 5%, in their chemical shower system in order to reduce the contact time to 2–4 min, followed by a 2–4 min water rinse. MCP is registered with the U.S. Environmental Protection Agency, and authorized by USDA for use in federally inspected meat and poultry plants.^16^ It has an extensive list of inactivation label claims against a number of gram-negative and gram-positive bacteria, fungi, and viruses.^17^ The list of viruses also includes both enveloped and non-enveloped viruses, including small non-enveloped viruses, the latter being the hardest ones to inactivate using chemical disinfectants.^18^ All the RG4 pathogens that are handled in BSL-4 laboratories are relatively large and enveloped viruses; hence, it was expected that MCP treatment would inactivate these viruses relatively easily, which is consistent with the scant published data available for MCP inactivation against RG4 viruses.^19–21^ However, whether the chemical shower process using 5% MCP can decontaminate a suit’s surface experimentally contaminated with a live virus has not been determined.

In addition, there are compliance requirements to national and international standards and guidelines that need to be met on various aspects of BSL-4 activities for a laboratory’s safe and ongoing operations. One such requirement is the need for each BSL-4 laboratory to undertake their own validations of various decontamination processes, including the protective suit decontamination in the chemical shower. For example, the WHO’s *Laboratory Biosafety Manual*,^22^ the U.S. Department of Health and Human Services’ *Biosafety in Microbiological and Biomedical Laboratories*,^2^ and the Public Health Agency of Canada’s *Canadian Biosafety Standard*^1^ require the suits be decontaminated while exiting the BSL-4 containment. Moreover, the *Laboratory Biosafety Manual*, 4th ed. includes guidance requirements on how to validate and document a decontamination: “in order to prove a material is decontaminated, laboratory personnel must validate the robustness of the decontamination method by measurement of the remaining biological agents against the detection limit by chemical, physical, or biological indicators.” Requirement 5.1.4 of the *Canadian Biosafety Standard*, 3rd ed. states “the performance of decontamination technologies is to be validated under in-use conditions using representative loads in conjunction with application-specific biological indicators, chemical integrators, and/or parametric monitoring devices consistent with the technology.” Also, these requirements need to be continuously met to maintain the license/certification status of BSL-4 laboratories with their national regulators, without which, a laboratory would not be allowed to operate.

Therefore, this study was undertaken to validate the effectiveness of a chemical shower decontamination process using MCP to mitigate the potential risk of pathogen escape from containment via potentially surface contaminated BSL-4 protective suits. Such validation is in support of compliance requirements for national/international standards/guidelines, as well as maintenance of our BSL-4 laboratory’s license/certification status with national regulators.

## Materials and Methods

### The Chemical Shower System

The chemical shower anteroom used in this study was 1.98 m wide, 1.98 m deep, and 2.89 m high; each corner of the room was equipped vertically with 1-inch stainless steel pipes (0.83-inch internal diameter). There were five thermoplastic spray nozzles (1/400-KY+H-KY6 Kynar Quick Fulljet^®^ Nozzles, 75° spray angle @20 psi, Spraying Systems Co. Wheaton, IL) installed per pipe, at 20 inches apart; the nozzles at the top were situated at 28 inches below the ceiling while the bottom ones were 7 inches above the floor. All 20 nozzles were aimed straight towards the center of the room to cover the BSL-4 positive pressure suit from top to bottom. The chemical, 5% MCP, was delivered at 18–20 psi through each nozzle at a rate of 3.14 L/min. The subsequent rinse phase, using tempered water, was delivered at approximately 40 psi at a rate of 5.3 L/min/nozzle. Thus, a 2 min chemical shower consumed 126 L of 5% MCP and the 3 min rinse consumed 318 L of water. Both MCP and water were delivered as a deluge to effect chemical decontamination and washing of the entire suit from top to bottom.

## Cell Line and Virus

African green monkey Vero E6 cell line (ATCC CRL-1586; American Type Culture Collection, Manassas, VA, U.S.) were maintained in Dulbecco’s modified Eagle’s medium (DMEM, Hyclone SH3024302) supplemented with 10% fetal bovine serum (FBS; Gibco 12484028) and 10 units/mL of penicillin/streptomycin (PS, Gibco 10378016), referred to henceforth as cell culture medium, and incubated at 37°C and 5% CO_2_. Vero E6 cultures involving virus replication were maintained in DMEM supplemented with 2% FBS and 10 units/mL of PS, referred to henceforth as virus culture medium Viral-Chlamydial-Mycoplasma Transport Medium (VCM), and incubated at 37°C and 5% CO_2_.

A stock of vesicular stomatitis Indiana virus (VSV) was prepared by infecting Vero E6 as described previously.^23^ Briefly, cell culture-flasks were infected with VSV at 0.001 multiplicity of infection and incubated for 3–4 days for the development of full viral cytopathic effect (CPE). Titers of the stocks were determined by the Reed–Muench procedure^24^ to be ≥8.8 log TCID_50_/mL. VSV Indiana is a RG2 pathogen of the *Rhabdoviridae* family.^25^ It is an enveloped virus that contains non-segmented, negative-sense single-stranded RNA genome similar to almost all the RG4 pathogens handled in BSL-4 laboratories. It grows well on Vero E6 cells and produces excellent CPE, which enables its detection and titration easy on cell culture under a light microscope.

### Preparation of Coupons

Our BSL-4 program was using positive pressure suits from two manufacturers, Dover Chemturion (ILC Dover, DE) and HVO (HVO-ISSI-Deutschland GmbH, Germany). Test coupons (approximately 3–6 cm^2^) were cut from decommissioned suits and included the transparent polyvinyl chloride visor material, suit fabric material, and rubber boot material. In addition, small coupons were made from vinyl tape material (floor marking blue tape, 1 inch wide, 8 mm thick, 3M Company MMM471-1X36YD-BL), which is used to attach the external gloves to the suit’s cuffs. All coupons were sterilized in-house by exposure to 1 mrads of gamma radiation using Gammacell excel (Cobalt-60 source).

**Figure 1.**
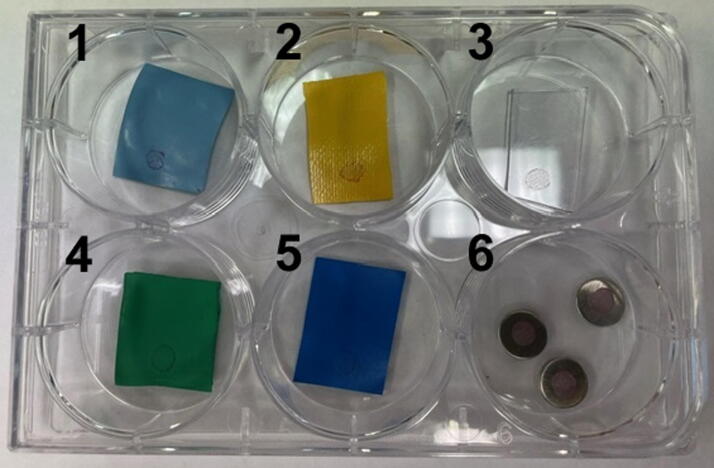
Preparation of test coupons for the BSL-4 chemical shower decontamination validation. Coupons made from Dover Chemturion suit material (1), HVO suit material (2), suit’s visor (3), suit’s rubber boot (4) and vinyl tape (5) deposited with 10 µL of dried viral inoculum are shown. Three steel carries with the inoculum in well (6) were used to determine the titer of the challenge virus. BSL-4, biosafety level 4.

**Figure 2.**
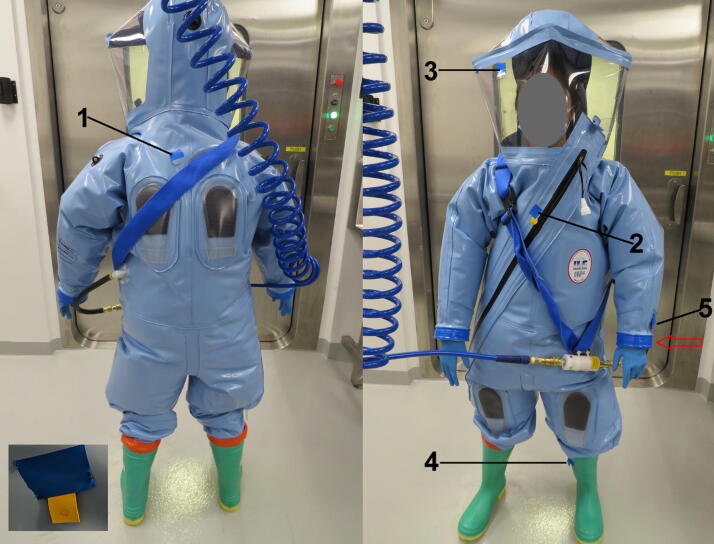
Placement of test coupons at various locations on a Dover Chemturion BSL-4 suit immediately prior to the chemical shower validation. A Dover suit coupon (1), HVO suit coupon (2), visor coupon (3), rubber boot coupon (4) and vinyl tape coupon (5) were attached to the suit. The arrow indicates the vinyl tape being used to attach external gloves to the suit’s cuffs. The inset shows how a test coupon is attached to the suit without affecting the dried-on virus inoculum. BSL-4, biosafety level 4.

To prepare VSV-laden test coupons, we followed an American Society for Testing and Materials (ASTM) International standard disinfectant testing method, ASTM E2197.^26^ The viral test inoculum was prepared by mixing VSV stock culture with a standardized organic tripartite soil load.^27^ Sterile coupons were placed in the wells of a 6-well plate, 10 µL of the VSV inoculum was deposited onto each coupon, and coupons were air-dried for 45–60 min in a biological safety cabinet (Figure 1). A fully trained BSL-4 worker donned a positive pressure suit, and coupons were attached using 3M vinyl tape at various locations on the suit’s external surface (Figure 2) so that the tape only stuck on the edge of the coupon away from the virus inoculum (inset, Figure 2). The worker then entered the chemical shower and initiated the chemical shower cycle, which involved 2 min of 5% MCP followed by 3 min of water rinse. Normal chemical shower procedures while exiting our BSL-4 laboratory involve the person vigorously scrubbing all areas of their suit’s external surfaces using gloved hands and pulling the excess suit material in the leg area upward to unfold and straighten the suit to ensure that chemical can contact the entire surface area. However, for this validation, the worker was asked not to scrub the suit, which could risk dislodging the attached coupons. Instead, the worker meticulously turned around and positioned themselves to ensure that all attached coupons came in contact with the chemical deluge and the subsequent water rinse.

Upon completion of the shower, the coupons were retrieved using sterile forceps and transferred to a new 6-well plate. The coupons were processed in a BSC under BSL-2 conditions; 2 mL of fresh VCM was added to each well, and the material was eluted by repeated pipetting of the coupons. All the eluted material was transferred to a new 6-well plate containing 80% confluent Vero E6 cells and topped up with an additional 2 mL of fresh VCM. The wells were examined over 5 days for CPE, indicated by the development of granular appearance, rounding, and sloughing, and scored as positive (CPE present) or negative (CPE absent). Negative results from the initial culture (P0) were sub passaged a second time, wherein 200 µL of P0 material was added onto a new 6-well plate containing 80% confluent Vero E6 cells with 4 mL of VCM (P1). Plates were incubated for an additional 5 days and scored for CPE to verify a true negative virus growth result.

The challenge virus titer was determined by adding 10 µL of virus inoculum to a triplicate set of stainless steel carriers (well 6, Figure 1) and allowed to dry for 1 h. The virus was eluted from steel carriers by adding 1 mL of VCM and repeat pipetting. The eluted virus was 10-fold serially diluted in VCM and plated on Vero E6 cells in replicates of 5 per dilution in a 96-well plate. Cultures were examined after 5 days, and dilutions were scored for CPE with titer calculated by the Reed–Muench procedure (Table 1).

**Table 1.** Summary of the virus recovery results from the “chemical showered” BSL-4 protective suit coupons inoculated with dried on VSV-organic soil load mixture

Description	Rep 1	Rep 2	Rep 3
P0			
Virus titer/coupon^a^	6.2 ± 0.8	6.2 ± 0.8	6.2 ± 0.8
Positive control	**+**	**+**	**+**
Negative control	−	−	−
Dover suit coupon	−	−	−
HVO suit coupon	−	−	−
Visor coupon	−	−	−
Boot coupon	−	−	−
Vinyl tape coupon	−	−	−
P1			
Positive control	**+**	**+**	**+**
Negative control	−	−	−
Dover suit coupon	−	−	−
HVO suit coupon	−	−	−
Visor coupon	−	−	−
Boot coupon	−	−	−
Vinyl tape coupon	−	−	−

^a^ TCID_50_ titer in log_10_ recovered from a triplicate set of stainless steel carriers that were inoculated with 10 µL VSV-soil load mixture/carrier, and then allowed to dry for 1 h. BSL-4, biosafety level 4; VSV, vesicular stomatitis virus.

A total of three chemical shower validation assays were conducted as described above; the first two were done over a period of 2 weeks, and the third one was performed a year later, with each shower consisting of a different worker per assay. A Dover Chemturion suit was used for the first and third runs, while an HVO suit was used for the second run.

## Results and Discussion

At the time of this study, our BSL-4 program was using positive pressure suits from Dover Chemturion and HVO-ISSI-Deutschland GmbH. Therefore, small test coupons were made only from Dover and HVO suits. In addition, test coupons from vinyl tape were also included for validation because vinyl tape was used for attaching the external gloves to the suit-cuffs (Figure 2). As shown in Figure 2, coupons were attached to the back, front, head (visor, forehead), arm (below elbow), and leg (below knee, on the inside) areas of the suit to validate the reach of the disinfectant chemical shower over the entire external surfaces of the suit to effect a thorough decontamination. Each test coupon was laden with more than 6 log TCID_50_ of VSV mixed with a standardized organic soil load; the virus inoculum was allowed to dry before the coupons were affixed to the suit for the disinfectant chemical shower testing. The tripartite matrix, which consisted of BSA, tryptone, and mucin represents an organic soil load similar to the secretions/excretions within which the virus is normally released from an infected person/animal^28^ and is incorporated into the challenge inoculum to increase the stringency of the test. The BSL-4 protective suit with the affixed test coupons was then subjected to a 2-min chemical shower using 5% MCP, followed by a subsequent water rinse for 3 min. Material eluted from all coupons was negative for viral growth (Table 1), indicating that the 2-min shower using 5% MCP followed by 3-min water rinse was sufficient to decontaminate the experimentally contaminated coupons and, by extension, a potentially contaminated suit. Previous studies have shown that the presence of MCP residues produced severe cytotoxicity on Vero cell cultures,^19,20^ which could complicate viral recovery assays in tissue culture. However, we observed no cytotoxicity in our cell cultures, proving that the 3-min water rinse was enough to remove all residual chemical from the suit.

Decontamination is defined as the process of freeing an object from harmful material;^29^ in this case, it means freeing of suit material coupons from VSV. This could happen through mechanical washing by the shower or by the MCP solution inactivating the virus, or likely a combination of both. While we haven’t differentiated between these mechanisms, two publications by Huang et al.^19,20^ suggest that 5% MCP solution likely inactivates the virus within 2 min.

A study by Park et al.^4^ showed the importance of physical scrubbing of the suit surface using a soft brush during the chemical shower, without which they could only achieve a less than 2 log reduction in bacterial spores (*Bacillus atrophaeus*) deposited onto the surface of a Dover Chemturion suit. In our experiment, we could not actively clean or scrub our test coupon surfaces without risking them being dislodged from the suit, but we did ensure that all coupons came in contact with the chemical disinfectant and subsequent water rinse. All test coupons that were heavily contaminated with dried on virus-soil load mixture were successfully decontaminated, showing more than a 6-log reduction. It is possible that the disinfectant used by Park et al., namely 0.2% Virkon (Antec International, Ltd., UK) and 0.25% Desintex (Laboratoires Rochex, France), did not have the cleaning capacity or surfactant concentration of the 5% MCP that we used for this study. It is also possible that the decontamination of a BSL-4 protective suit experimentally contaminated with bacterial spores is much more challenging than that contaminated with an enveloped virus such as VSV.

In the best scenario, a BSL-4 chemical shower validation should be performed using a protective suit experimentally contaminated with one or more high-consequence RG4 viruses, such as Ebola. However, due to the extreme safety risks involved with such an experimental design, we opted for a safer, surrogate RG2 virus, specifically VSV. VSV, an enveloped RNA virus, has been used as a surrogate virus for studying Ebola virus inactivations.^21,23^ VSV is structurally and biochemically similar to most of the high-consequence viruses that are handled in the BSL-4 laboratories and it replicates relatively rapidly to high titers on Vero E6 cells^30^ with pronounced CPE, making it an ideal surrogate virus for RG4 viruses. While VSV was a good choice, future studies could benefit from including even tougher, non-enveloped viruses like parvoviruses or picornaviruses as potential surrogates. This would be especially valuable for response readiness to handle emerging viral threats.^31^

Even though 5% MCP was effective in decontaminating an experimentally contaminated BSL-4 protective suit during the chemical shower process, one of the chemicals in MCP, nonylphenol ethoxylate, is highly toxic to the environment and has been banned in many European countries. Therefore, some BSL-4 laboratories in Europe have been using liquid PAA in the chemical shower to decontaminate their protective suits. PAA is an excellent microbicide; it is environmentally friendly and breaks down to water, oxygen, and acetic acid.^28^ However, liquid PAA is corrosive; it reacts with a variety of organic compounds, and acute and chronic health effects on workers have been reported when they were exposed to even below the occupational exposure limits of 0.2 ppm.^32,33^ While BSL-4 protective suits offer protection from particulates and aerosols, they are not necessarily made to offer protection from chemical vapors. Hence the use of liquid PAA in BSL-4 chemical showers could potentially expose workers to PAA vapors resulting in unintended health consequences.

QAC-based disinfectants, even in the absence of nonylphenol ethoxylates, are facing increased scrutiny because of their harmful impacts on the environment and human health.^34^ Therefore, more research is needed to identify suitable, ideally non-QAC-based, chemical shower disinfectants that are effective against the high-consequence pathogens handled in BSL-4, are safe to the workers and to the environment, have a relatively long shelf life when diluted, and are compatible with the shower delivery facility infrastructure. The deluge shower system validated in this study consumed 126 L of 5% MCP per shower, but attempts should be made to evaluate other shower delivery system options that might reduce MCP consumption in the meantime.

## Conclusion

We used BSL-4 protective suit coupons inoculated with dried-on high titer VSV-organic soil load mixture and affixed them onto either a Dover Chemturion suit or an HVO suit to validate the efficacy of our BSL-4 chemical deluge shower system. Mixing the test virus with the soil load and drying the mixture on to the suit coupons makes them much harder for the chemical disinfectant shower to decontaminate; however, all the test coupons from all three validation trials were successfully decontaminated, resulting in no detectable virus. The fact that all the coupons were decontaminated, without subjecting them to mechanical scrubbing, shows the thoroughness of our deluge chemical shower system, which included 2 min of a 5% MCP shower followed by 3 min of a water rinse. Foregoing mechanical scrubbing during the validation runs shows that our validation testing method was more stringent than the routine shower out process, where our personnel leaving BSL-4 laboratory scrub their suit surfaces using gloved hands both during the chemical and water phases of the shower process. MCP contains chemicals that are toxic to the environment, and more studies are needed to find a suitable environmentally friendly disinfectant to replace MCP in BSL-4 laboratories across the world.
